# Band structure regulation in Fe-doped MgZnO by initial magnetic moments†

**DOI:** 10.1039/d0ra09306h

**Published:** 2021-01-14

**Authors:** Licheng Zheng, Qi Yao, Hao Wang, Huahan Zhan, Wenwei Cai, Yinghui Zhou, Junyong Kang

**Affiliations:** Fujian Provincial Key Laboratory of Semiconductor Materials and Applications, CI Center for OSED, College of Physics Science and Technology, Xiamen University Xiamen 361005 P. R. China h_wang@xmu.edu.cn huahan@xmu.edu.cn; Research Institute for Biomimetics and Soft Matter, Xiamen University Xiamen 361005 P. R. China

## Abstract

ZnO-based diluted magnetic semiconductors have high prospects in spintronics applications. In this study, the electronic and magnetic properties of Fe-doped MgZnO are studied by density functional theory calculations. The investigations of the band structure, total density of states, and projected density of states revealed a strong correlation between Mg and O atoms in addition to the magnetism and impurity level generated by the Fe atoms. In the spin charge density and band structure of 2.78% Fe-doped MgZnO, Fe atoms always cause paramagnetic coupling with oxygen atoms bonded around them, and when the initial magnetic moments were parallel, the band gap is broadened in the opposite channel. On the contrary, when the initial magnetic moments are anti-parallel, the band gap is narrowed in both the spin-up and spin-down channels. This shows that the initial magnetic moments have a great influence on the band structure, giving another way to tune the gap dynamically.

## Abstract 2

The optoelectronic and magnetic properties of Fe-doped MgZnO are regulated by the Fe dopant and its initial magnetic moments.

## Graphical abstract

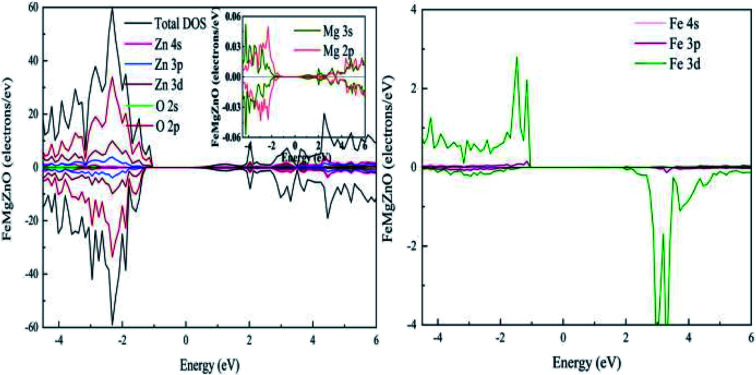

## 1. Introduction

Diluted magnetic semiconductors (DMSs) have a wide range of application prospects in optoelectronics and spin devices, hence attracting increasing attention. They are usually fabricated by incorporating transition elements, particularly the ferromagnetic elements such as Fe, Co and Ni, into non-ferromagnetic semiconductors.^1–4^

The electronic and magnetic properties of DMSs are tuned by the concentration of transition metal dopants and further regulated by spin injection.^5–8^ Take GaMnAs for example, a Curie temperature as high as 318 K has been achieved with a mole composition of Mn around 5%.^9^ However, for many diluted magnetic semiconductors, the Curie temperature is usually far below room temperature, which greatly limits their application scenarios. Therefore, researchers prefer semiconductors, such as ZnO, GaN, and In_2_O_3_, with natural Curie temperatures exceeding room temperature.^2^

In recent years, ZnO-based DMS has attracted great attention. This is because ZnO is a wide direct band gap (3.37 eV) semiconductor with unique physical and chemical properties, particularly a large exciton binding energy (60 MeV) at room temperature, and other advantages such as environment friendliness, availability of different preparation methods, relatively low cost.^10,11^

The application scenario for ZnO-based materials is greatly broadened by employing the band engineering process and the incorporation of transition metals. For Fe-doped MgZnO, the band gap can be modulated between 3.37 eV (ZnO) and 7.80 eV (MgO) by changing the composition of Mg.^12^ The addition of Fe not only further regulates its electronic properties, but also endows the material with unique magnetic properties. Thus, it is interesting and important to study the electronic and magnetic properties of ZnO-based DMS experimentally and theoretically, which have been seldomly reported.^13,14^

With this consideration, herein, the first-principle calculations are employed to study Fe-doped MgZnO. In 1Fe-doped MgZnO, the most stable configuration employed for further investigation was selected by determining the total energy, which is the lowest when the Fe atom occupies the second nearest site at the same A-face of the Mg atom. The correlation among different atoms was analyzed by the total and projected density of state. In the case of 2Fe-doped MgZnO, the spin charge density of different initial magnetic moments is used to analyse the influence of Fe atoms on the surrounding atoms. Moreover, the band structure of the spin-up and spin-down channels of the two Fe atoms were calculated to analyse the effects of different initial magnetic moments. We showed that the initial magnetic moments have a great influence not only on the total magnetic moment but also on the band structure, giving another way to tune the electronic and magnetic properties dynamically.

## 2. Calculation method and model

The first principle calculations presented in this study were carried out by Vienna *Ab initio* Simulation Package (VASP) on the basis of density functional theory (DFT).^15–17^ The electron exchange and interactions are described by the generalized gradient approximation (GGA), while the electron–ion interaction is described by the Perdew, Burke and Ernzerhof (PBE) method.^16,18,19^ The energy cut-off is 500 eV. After each cycle, the adopted conjugate gradient algorithm was stopped when the energy difference became less than 10^−5^ eV and the stress difference per atom was less than 0.01 eV Å^−1^. Considering that the calculations with a standard GGA cannot correctly describe the strongly correlated systems with partially filled d or f shells, the GGA+U strategy was adopted in all calculations, which was practicable for on-site Coulomb interactions within the Dudarev approximation.^20,21^ The value of *U* was set to 3.8 eV for the d-electrons of Zn atoms, although the band gap was still smaller than the experimental values and the lattice constant was more consistent with the experimental results (as shown in Table 1 below). Also, the value of *U* was set to 3.5 eV for the d-electrons of Fe atoms.^22^ The Brillouin zone is a 3 × 3 × 3 gamma-centered *K*-point mesh in the Monkhorst–Pack scheme.^23^

**Table 1:** The lattice parameters of ZnO, MgZnO, FeMgZnO and ZnO (experimental)

	ZnO	MgZnO	FeMgZnO	ZnO (experimental)^24^
*a* = *b* (Å)	3.247	3.247	3.249	3.249
*c* (Å)	5.229	5.224	5.226	5.205
*c*/*a*	1.610	1.609	1.608	1.602
*α* = *γ* (°)	90	90	90	90
*β* (°)	120	120	120	120

The crystal structure of ZnO used for the calculations was the wurtzite structure (space group *C*6*v*4–*P*6_3_*mc*). The Zn atom and the four nearest neighbor O atoms constitute a tetrahedron. The Zn atom is at the center of the tetrahedron and it hybridized the d orbital with the p orbital of the O atoms. Along the (0001) direction, O atoms and Zn atoms were alternately stacked layer by layer to form a ZnO lattice.

**Fig. 1:**
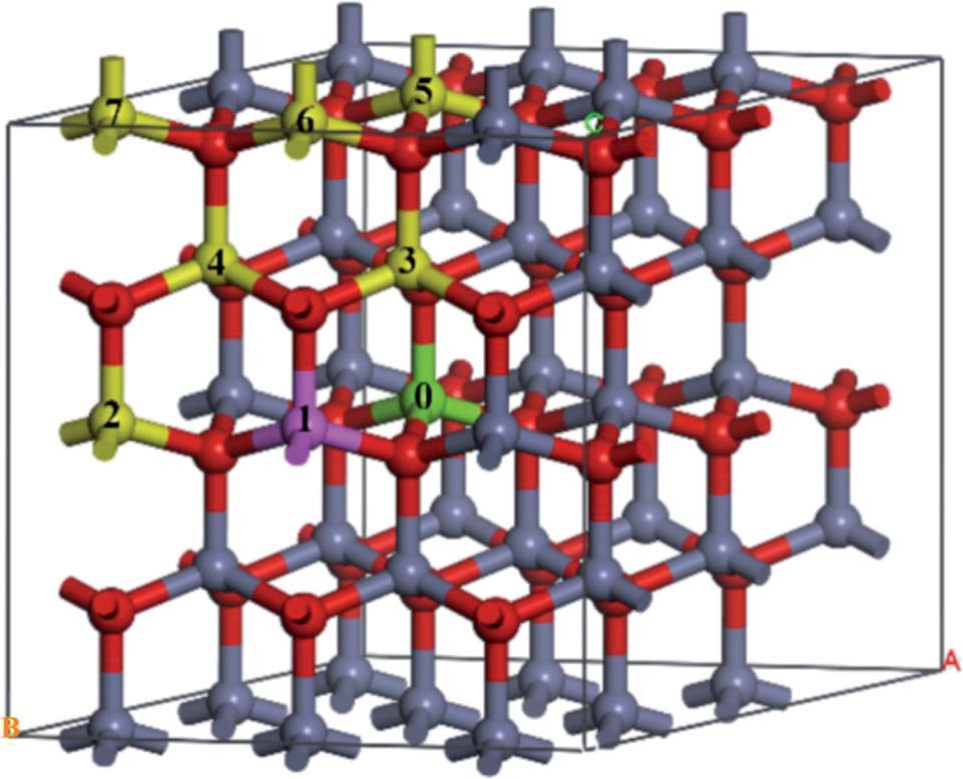
The structure of Fe-doped MgZnO. The site 0 for Mg atoms, and 1–7 represent candidate sites for doping with Fe atoms.

A 3 × 3 × 2 supercell was used, as shown in Fig. 1. The Mg atom is put at the center of the A-face of the supercell (site 0) and the Fe atoms are placed at the following sites, including the same A-face (site 1: the nearest atom, site 2: the second nearest atom), the nearest B-face (site 3: the nearest atom, site 4: the second nearest atom), and the nearest A-face (site 5: the nearest atom, site 6: the second nearest atom, site 7: the third nearest atom).

## 3. Results and discussion

### 3.1 Structural properties

Crystal structure is often modified after the incorporation of dopant atoms. The lattice constants of ZnO, MgZnO and Fe-doped MgZnO (FeMgZnO) after structural optimization are shown in Table 1. Compared to the experimental results, the lattice constants of ZnO, *a* and *b* were slightly smaller, while *c* was a little bit bigger. Compared to those in ZnO, *c* decreased, while *a* and *b* remained almost unchanged in MgZnO, but *a* and *b* increased, while *c* and *c*/*a* also reduced in FeMgZnO.

**Fig. 2:**
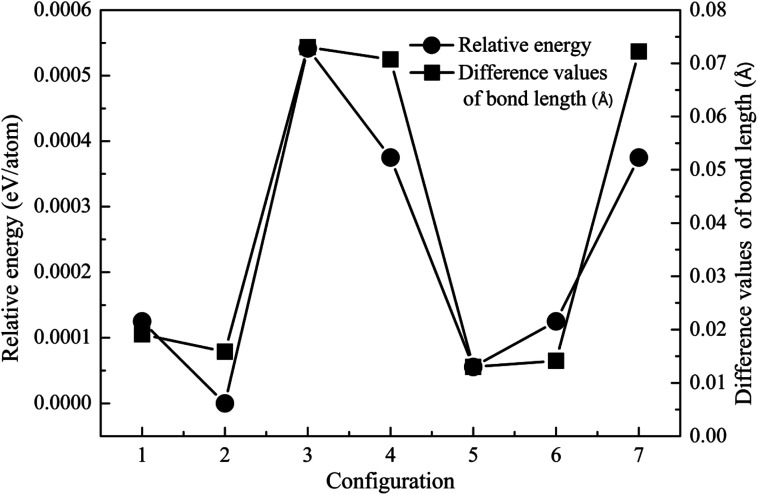
The relative energy (cycle line) and difference in the bond length (square line) of the 1Fe-doped MgZnO.

In the following investigation, for the case of 1Fe-doped MgZnO, the location of the Fe atom (denoted as Fe1) was selected by comparing the system total energies with Fe1 at different sites (sites 1–7), as shown in Fig. 2. As a result, Fe1 located at site 2 (configuration 2) was adopted for further analysis due to its lowest relative energy.

### 3.2 Electronic properties

**Fig. 3:**
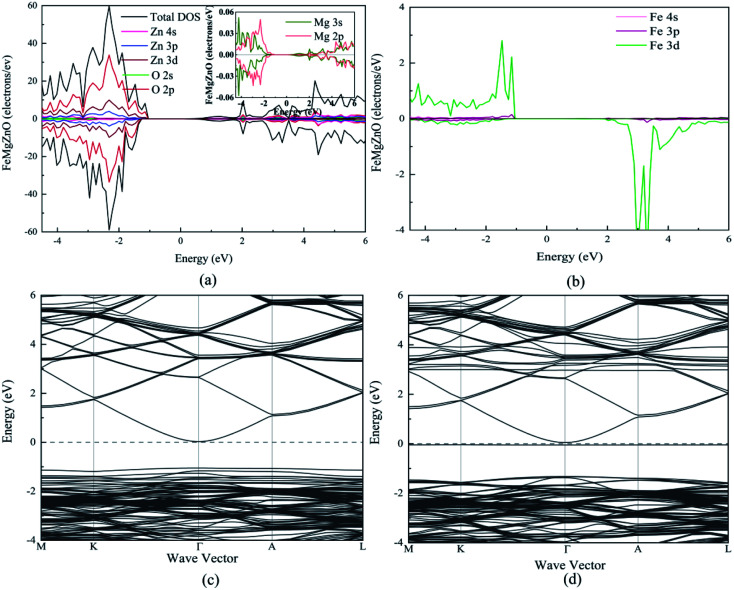
The TDOS, PDOS and band structure of 1Fe-doped MgZnO. (a) The TDOS and PDOS of Zn and O, and the PDOS of Mg in the inset figure, (b) the PDOS of Fe, (c) the band structure of the spin up channel and (d) the band structure of the spin down channel.

For 1Fe-doped MgZnO, the electronic properties were demonstrated by the total and projected density of states (TDOS and PDOS), and the band structure, as depicted in Fig. 3.

It can be seen from the TDOS and PDOS of Zn, O and Mg in Fig. 3a that the conduction band of 1Fe-doped MgZnO is mainly composed of O 2p and Zn 4s orbits, while the valance band is mainly composed of O 2p and Zn 3d orbits. The PDOS of Mg is mainly composed of 2p and 3s orbits. Compared with the PDOS of oxygen, the Mg 3s and O 2p orbits in the conduction band are similar as well as the Mg 2p and O 2p orbits in the valence band are similar. This shows a strong correlation between Mg and O. Besides, TDOS is asymmetric, implying that the magnetism is generated.

As shown in Fig. 3b, the PDOS of Fe is mainly composed of 3d orbit and extreme asymmetry was observed in both the conduction band and the valence band, indicating that Fe is the main cause of magnetism.

The band structure of 1Fe-doped MgZnO is shown in Fig. 3c and d. Both the conduction band minimum (CBM) and the valence band maximum (VBM) are located at the *Γ* point, demonstrating a direct band gap, and Fe introduced impurity levels in the spin-down channel near the Fermi level and CBM. Thus, for 1Fe-doped MgZnO, the asymmetry of Fe's PDOS and the strong correlation between Mg–O bring significant changes in the electronic properties, which is of great importance for changing the electronic structure of the ZnO-based materials.

### 3.3 Influence of the initial magnetic moments on the total magnetic moment

**Fig. 4:**
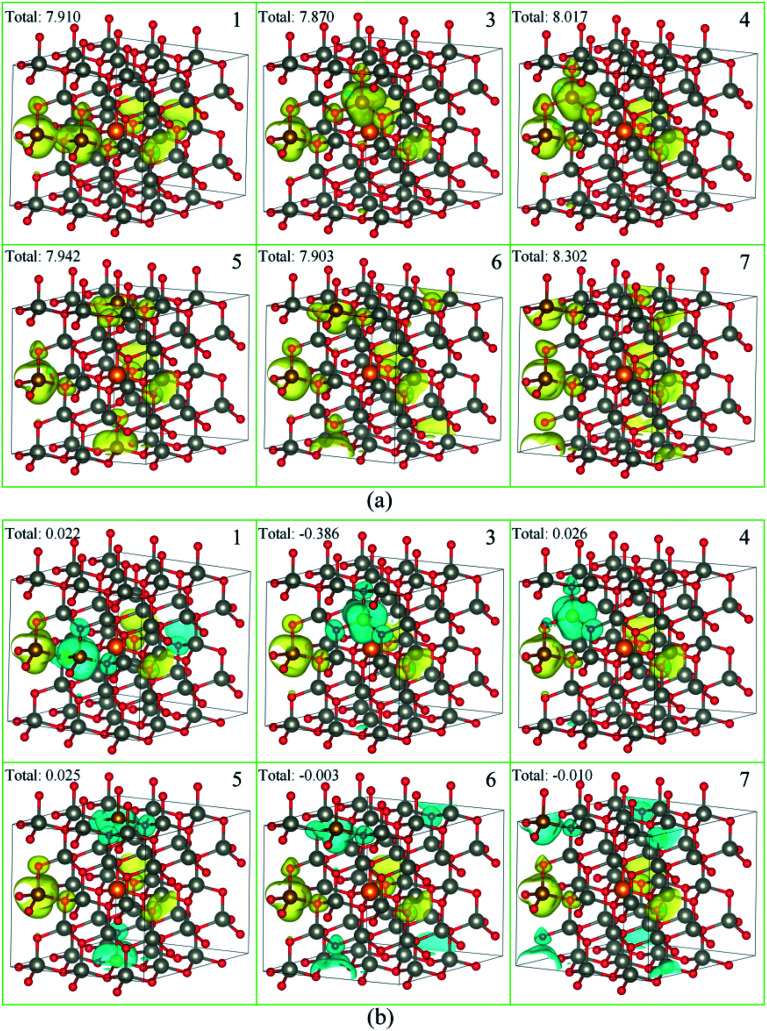
The spin charge density of (a) ↑↑, (b) ↑↓, yellow represents spin-up density, and cyan represents spin-down density.

As a ferromagnetic element, the Fe atoms not only cause the significant modulation in the structural and the electronic properties of ZnO materials but also cause the significant characteristic of spin injection. In order to analyze the effects of different initial magnetic moments of the Fe atoms, the doping of the second Fe atom (denoted as Fe2) was simulated by fixing the first Fe atom (denoted as Fe1) at site 2, then Fe2 was made to replace Zn atom at sites 1, 3–7 respectively. The further calculation is divided into two parts. First, the initial magnetic moments of the two Fe atoms are set to spin-up parallel (marked as ↑↑). Secondly, the initial magnetic moments of the two Fe atoms are set to anti-parallel (marked as ↑↓, ↑ for Fe1, ↓ for Fe2). The spin charge densities under different configurations are shown in Fig. 4.

Again, it was proved that the doped Fe atoms are the main cause for the magnetic moments generated in the system. The magnetic moment of O atoms bonded with the Fe atoms was largely affected by the Fe atoms and exhibited paramagnetic coupling, while the magnetic moments of Mg atoms and other atoms were almost unaffected. When the initial magnetic moments are parallel, the total magnetic moment is greatly increased. This indicated that the parallel initial magnetic moments of the Fe atoms resulted ferromagnetic coupling in the system. However, when the initial magnetic moments were anti-parallel, the total magnetic moment was negligible because the magnetic moment was largely neutralized, leading to the occurrence of the anti-ferromagnetic coupling phenomenon.

When Fe2 is in site 1 or site 5 (configuration 1 or 5), one of the O atoms bonded with two Fe atoms. For this O atom, when the initial magnetic moments are parallel, its magnetic moment is about twice that of O atoms that were bonded with only one Fe atom. However, when the initial magnetic moments were anti-parallel, its magnetic moment was almost negligible. This further shows that the initial magnetic moments of the Fe atoms greatly affect the O atoms bonded with them and exhibits paramagnetic coupling.

### 3.4 Regulation of the band structure by the initial magnetic moments

**Fig. 5:**
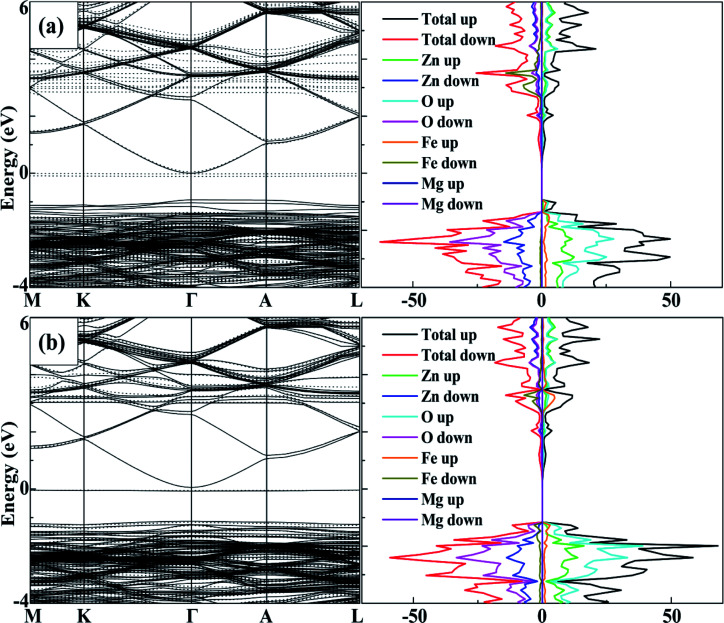
The band structure and density of state of configuration 1, the spin-up (solid line) and the spin-down (dotted line) channels and the corresponding density of state of 2Fe-doped MgZnO with (a) ↑↑, (b) ↑↓.

To analyse the tuning effects of initial magnetic moments, the band structures of 2Fe-doped MgZnO with different configurations were also calculated. The band structure and density of state of configuration 1 is plotted in Fig. 5a for the cases with initial magnetic moments set to ↑↑, and Fig. 5b for those set to ↑↓. It can be seen that the CBM and VBM of 2Fe-doped MgZnO are located at the *Γ* point and remained as a direct band gap semiconductor.

In Fig. 5a, where the initial magnetic moments are ↑↑, the impurity levels associated with two Fe atoms appeared near the Fermi level in the spin-down channel. On the other hand, in Fig. 5b, where the initial magnetic moments are ↑↓, the impurity levels associated with the two Fe atoms appeared near the Fermi levels in both the spin-up and spin-down channels. Thus, for each Fe atom, the impurity level is present in the opposite channel of the initial magnetic moments. Consequently, in 2Fe-doped MgZnO with ↑↑, only the band structure of the spin-down channel was needed to be investigated, while in the case with ↑↓, both spin-up and spin-down channels were needed to be considered.

Thus, the band gap of 2Fe-doped MgZnO of each configuration with ↑↑ and ↑↓ initial magnetic moments are calculated and presented in Table 2, where the corresponding magnetic moments of 2Fe-doped MgZnO are also presented. Considering the spin-down channel in the case of ↑↑, even the band gap minimum (1.42 eV) was wider than that of the 1Fe-doped MgZnO (1.37 eV) and the MgZnO (1.32 eV). In the case of ↑↓, considering both spin-up and spin-down channels, even the band gap maximum (1.24 eV) was narrower than that of pure ZnO (1.26 eV). This shows that the different initial magnetic moments of the Fe atoms can severely affect the band structure. When parallel initial magnetic moments are set, the band gap is found to widen in the opposite channel. On the contrary, when the anti-parallel initial magnetic moments are set, the band gap is found to be narrowed in each channel.

**Table 2:** The band gap (eV) of 2Fe-doped MgZnO

Configuration	Spin setting	Spin up	Spin down
1	↑↑	0.93	1.42
	↑↓	1.18	1.22
3	↑↑	1.05	1.44
	↑↓	1.18	1.18
4	↑↑	0.97	1.45
	↑↓	1.22	1.24
5	↑↑	1.10	1.45
	↑↓	1.16	1.15
6	↑↑	1.06	1.45
	↑↓	1.16	1.15
7	↑↑	1.05	1.44
	↑↓	1.19	1.18

### 3.5 Impact of the *U* parameter of Zn on the calculation

**Fig. 6:**
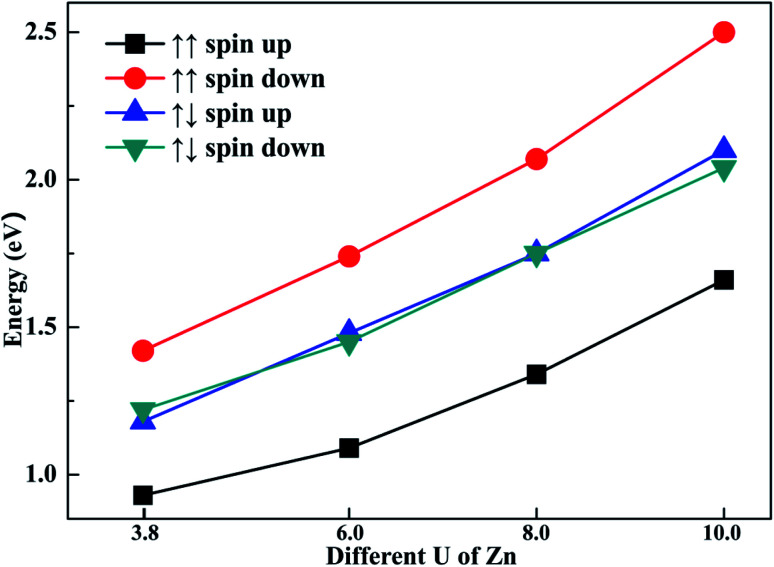
The different *U* parameters of Zn and their corresponding band gaps.

To explore the impact of different *U* parameters on the calculation, we also calculated the band structure and density of state of configuration 1 with different *U* parameters of Zn. In Fig. 6, the band gaps of different spin channels are presented. It is shown that the band gap widened with the increase in *U* parameter. Similar to the results calculated by *U* = 3.8, when the initial magnetic moments are ↑↑, the band gap calculated with different *U* values also widened in the opposite channel. Also, when the initial magnetic moments were ↑↓, the band gap as calculated from different *U* values also narrowed in both channels. That is, although the selection of *U* parameters may change the value of band gaps, the regulation effects of the different initial magnetic moments to band gap are similar. The above-mentioned investigation shows that the band gap can be tuned through different initial magnetic moments of the doped Fe atoms.

## 4. Conclusions

In this study, the electronic and magnetic properties of Fe-doped MgZnO are studied by VASP on the basis of DFT. It is seen in 1Fe-doped MgZnO that the system is the most stable when the Fe atom occupies the second nearest site at the same A-face of the Mg atom. Typical magnetism is introduced by the Fe dopant, and a strong correlation between the Mg and O atoms is depicted. The investigation of the spin charge density of different initial magnetic moments revealed that the oxygen atoms around the Fe atoms always demonstrate a similar spin polarization. Moreover, when the initial magnetic moments of the two Fe atoms are parallel, the band gap of the opposite channel is broadened; on the other hand, when the two Fe atoms are anti-parallel, the band gap of spin up and down channels are narrowed, showing significant characteristics of DMS, and the regulation effect of Fe-doping on MgZnO.

## Conflicts of interest

There are no conflicts to declare.

## Supplementary Material

RA-011-D0RA09306H-s001
