# Supplementary material for: Band structure regulation in Fe-doped MgZnO by initial magnetic moments
Source: RSC Adv. 2021 Jan 14;11(6):3209–15. doi: 10.1039/d0ra09306h (PMC8693980; doi:10.1039/d0ra09306h)
Supplement: RA-011-D0RA09306H-s001 [file RA-011-D0RA09306H-s001.pdf]

Electronic Supplementary Material (ESI) for RSC Advances.
This journal is © The Royal Society of Chemistry 2020

Band structure regulation in Fe-doped MgZnO by initial magnetic moment

Licheng Zheng[1], Qi Yao[1], Hao Wang[1, 2,*] Huahan Zhan[1,*] Wenwei Cai[1], Yinghui Zhou[1], Junyong Kang[1]

[1]*Fujian Provincial Key Laboratory of Semiconductor Materials and Applications, CI Center for OSED, College of Physics Science and Technology, Xiamen University, Xiamen 361005, P.R. China*

[2]*Research Institute for Biomimetics and Soft Matter, Xiamen University, Xiamen 361005, P.R. China*

S1 The comparison of relative energies of different configurations

In order to analyze the effect of different initial magnetic moments of the Fe atoms, the doping of the second Fe atom is simulated by fixing the first Fe atom at site 2, then Fe2 replaces the Zn atom at sites 1, 3-7 respectively. When the initial magnetic moments are anti-parallel, the total energy is lower as shown in Figure S1, thus more favorable.

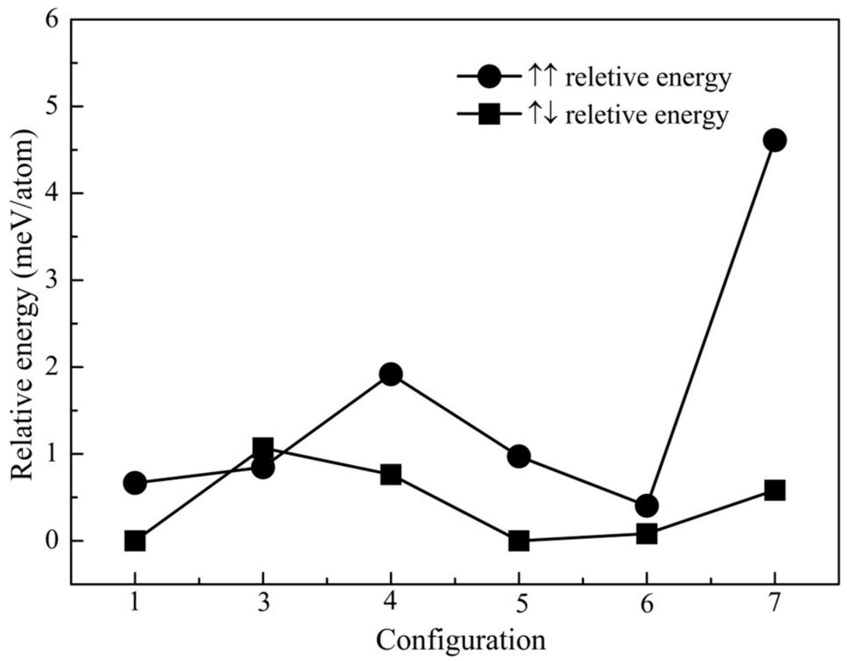

Figure S1 The comparison of relative energies of different configurations.

S2 The comparison of 3×3×3 and 5×5×5 sampling for K-points

In our work, 3×3×3 sampling for K point has been used. For comparison, we have recalculated the configuration 1 in the case of parallel initial magnetic moment with 5×5×5 sampling, and the comparison of the opposite channel is showed in Table S1, the difference of average energy eV/atom is around 1 percent, and those of total magnetic moments, band gap are less than 0.2 and 0.3 percent, respectively. This shows that 3x3x3 sampling for K point might be enough.

Table S1 The comparison of 3×3×3 and 5×5×5 sampling for K-points.

	Average energy (eV/atom)	Total magnetic moment (μB)	Band gap (eV)

3x3x3	-4.53	7.910	1.4212
5x5x5	-4.59	7.898	1.4171
Deviation	1.324%	0.152%	0.288%

S3 Band structure and density of state of each configuration

In addition to Figure 5 for configuration 1, here we present all of the band structure and density of state of each configurations in Figure S2-S7 for configuration 1, 3, 4, 5, 6, 7, respectively.

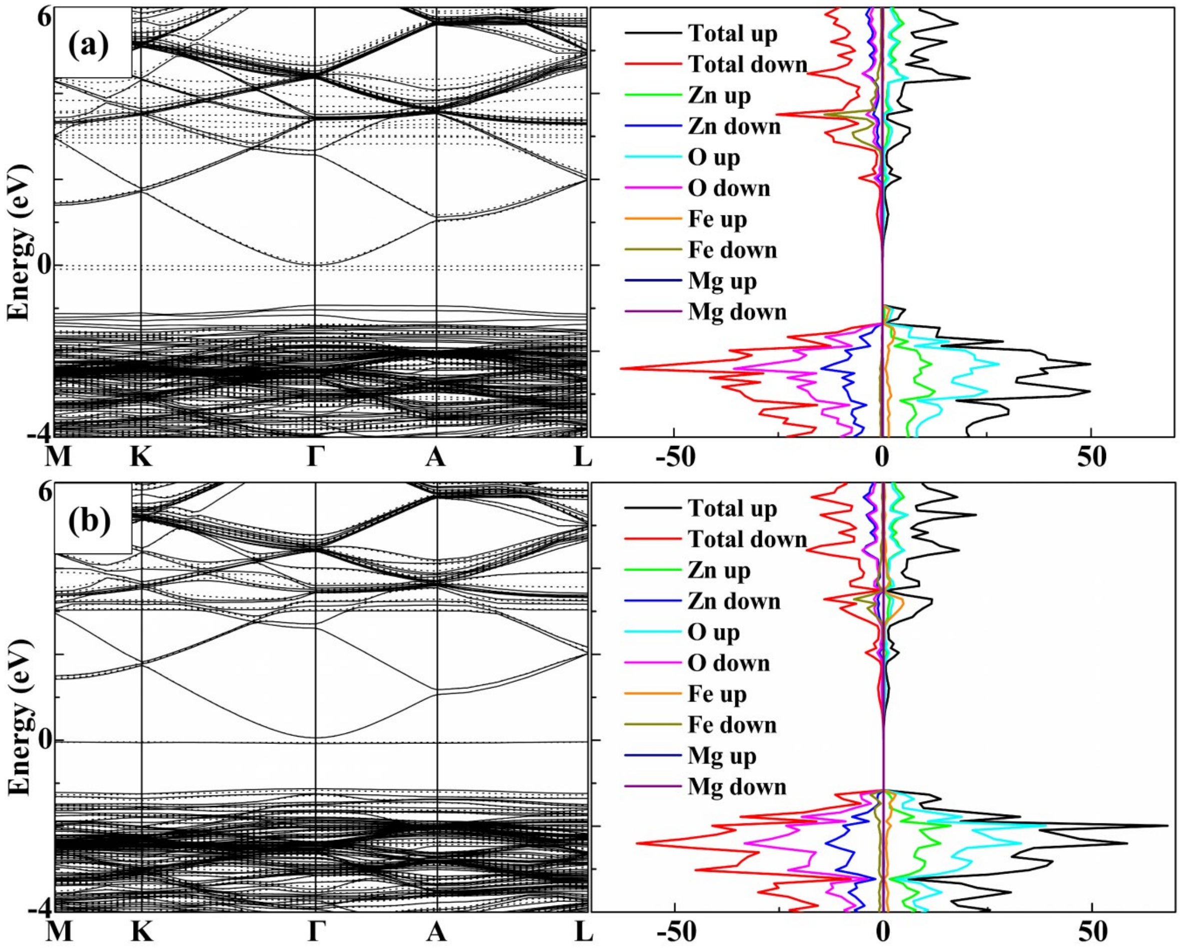

Figure S2. Band structure and density of state of configuration 1, the spin-up (solid line) and the spin-down (dotted line) channels and the corresponding density of state of 2Fe-doped MgZnO with (a) ↑↑, (b) ↑↓.

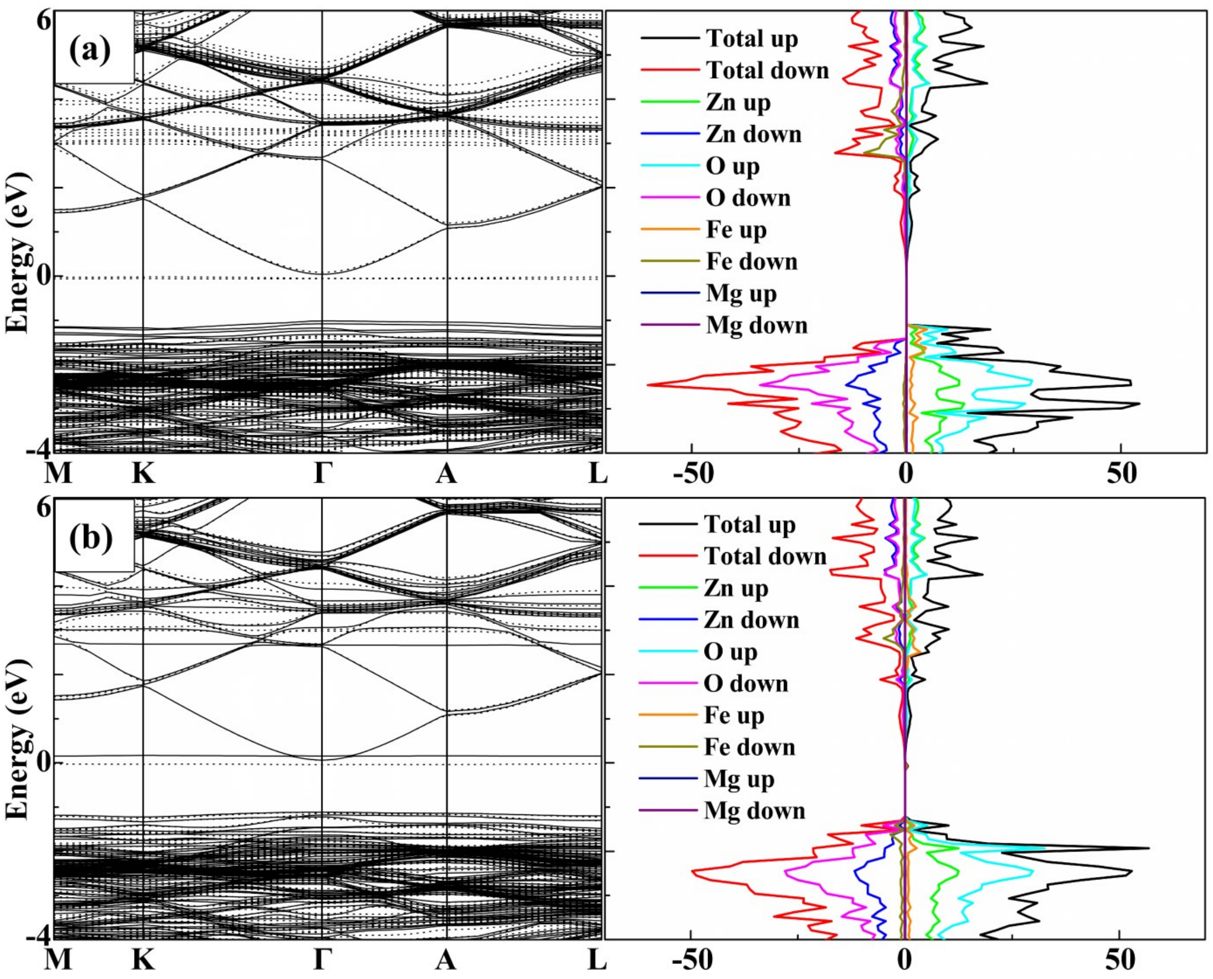

Figure S3. Band structure and density of state of configuration 3, the spin-up (solid line) and the spin-down (dotted line) channels and the corresponding density of state of 2Fe-doped MgZnO with (a) ↑↑, (b) ↑↓.

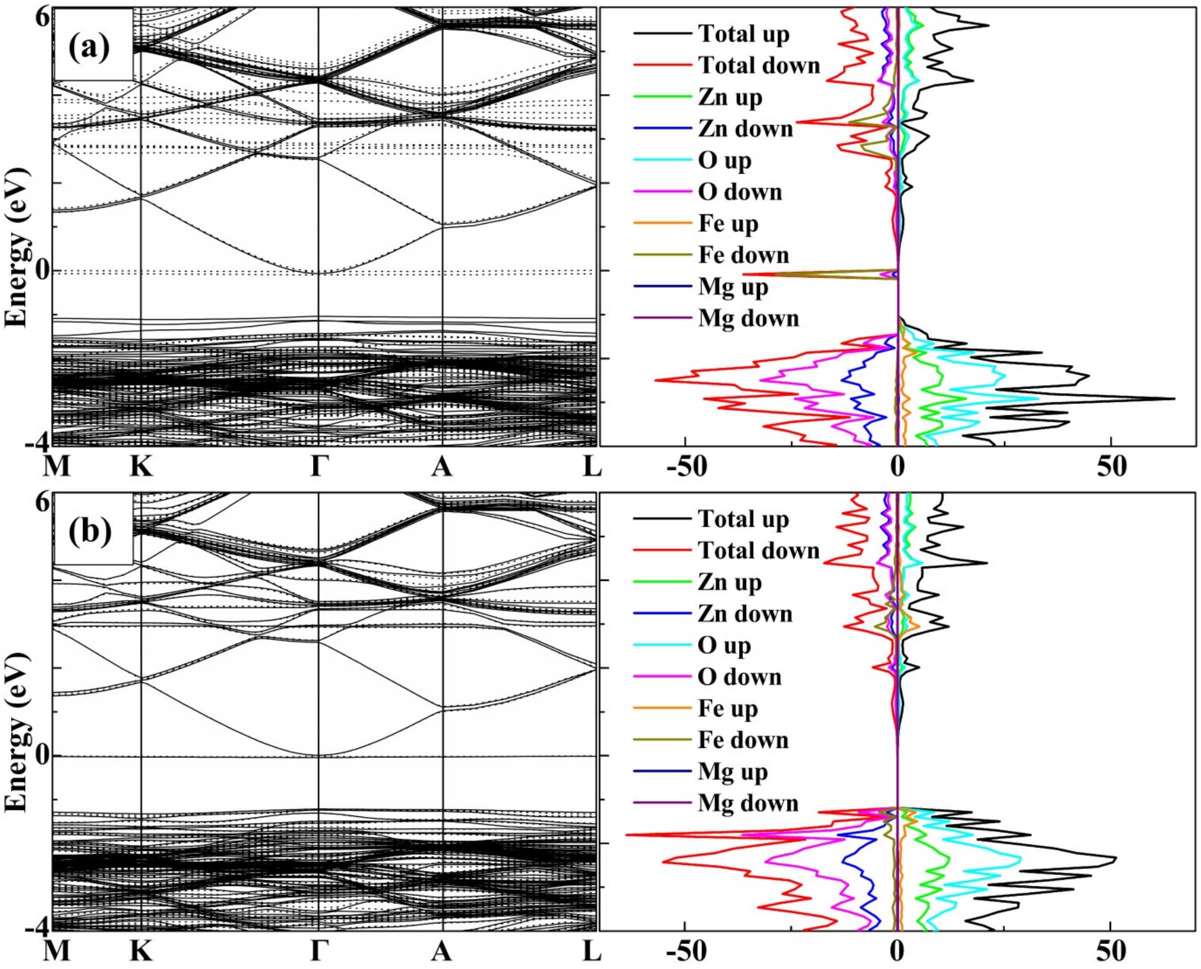

Figure S4. Band structure and density of state of configuration 4, the spin-up (solid line) and the spin-down (dotted line) channels and the corresponding density of state of 2Fe-doped MgZnO with (a) ↑↑, (b) ↑↓.

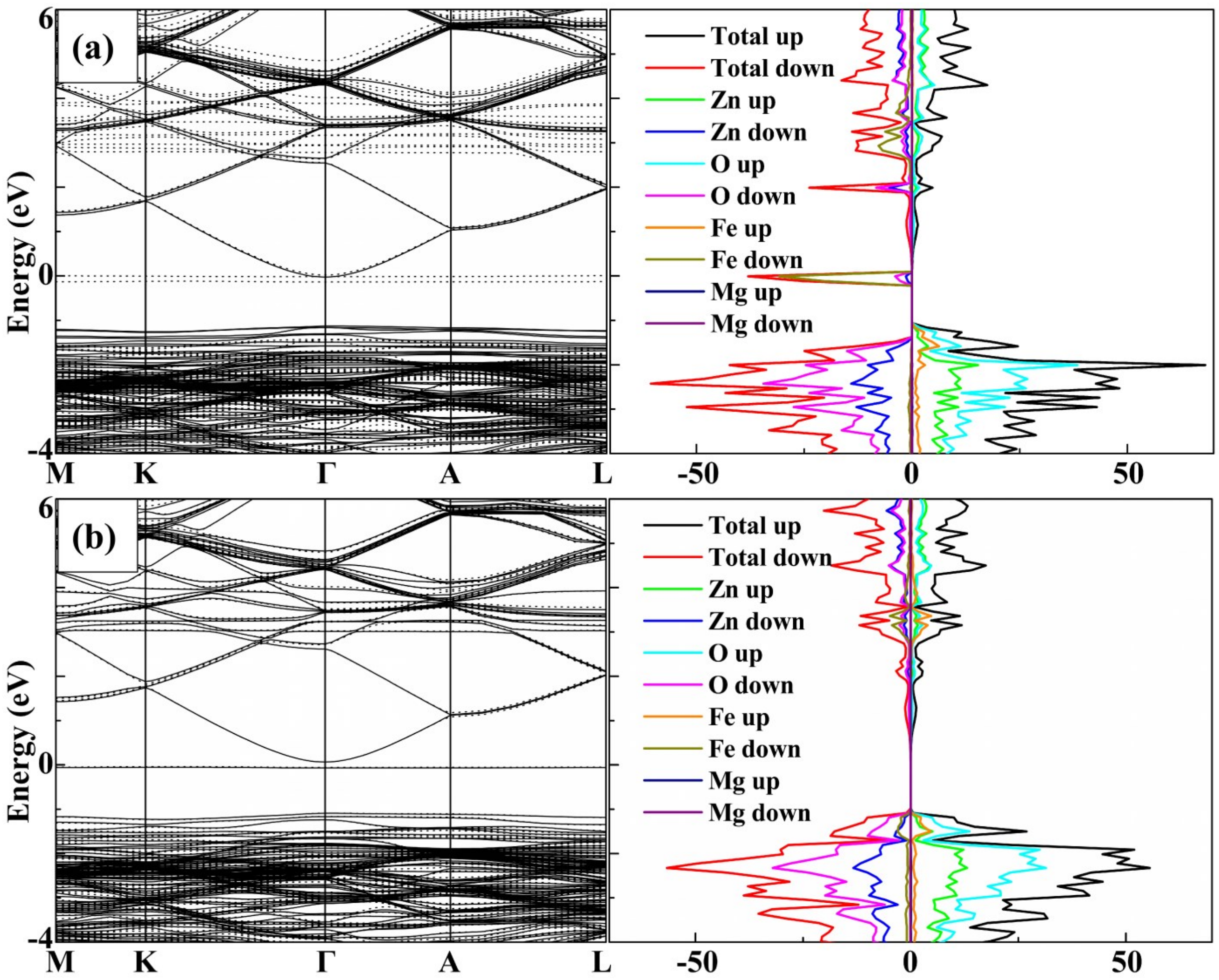

Figure S5. Band structure and density of state of configuration 5, the spin-up (solid line) and the spin-down (dotted line) channels and the corresponding density of state of 2Fe-doped MgZnO with (a) ↑↑, (b) ↑↓.

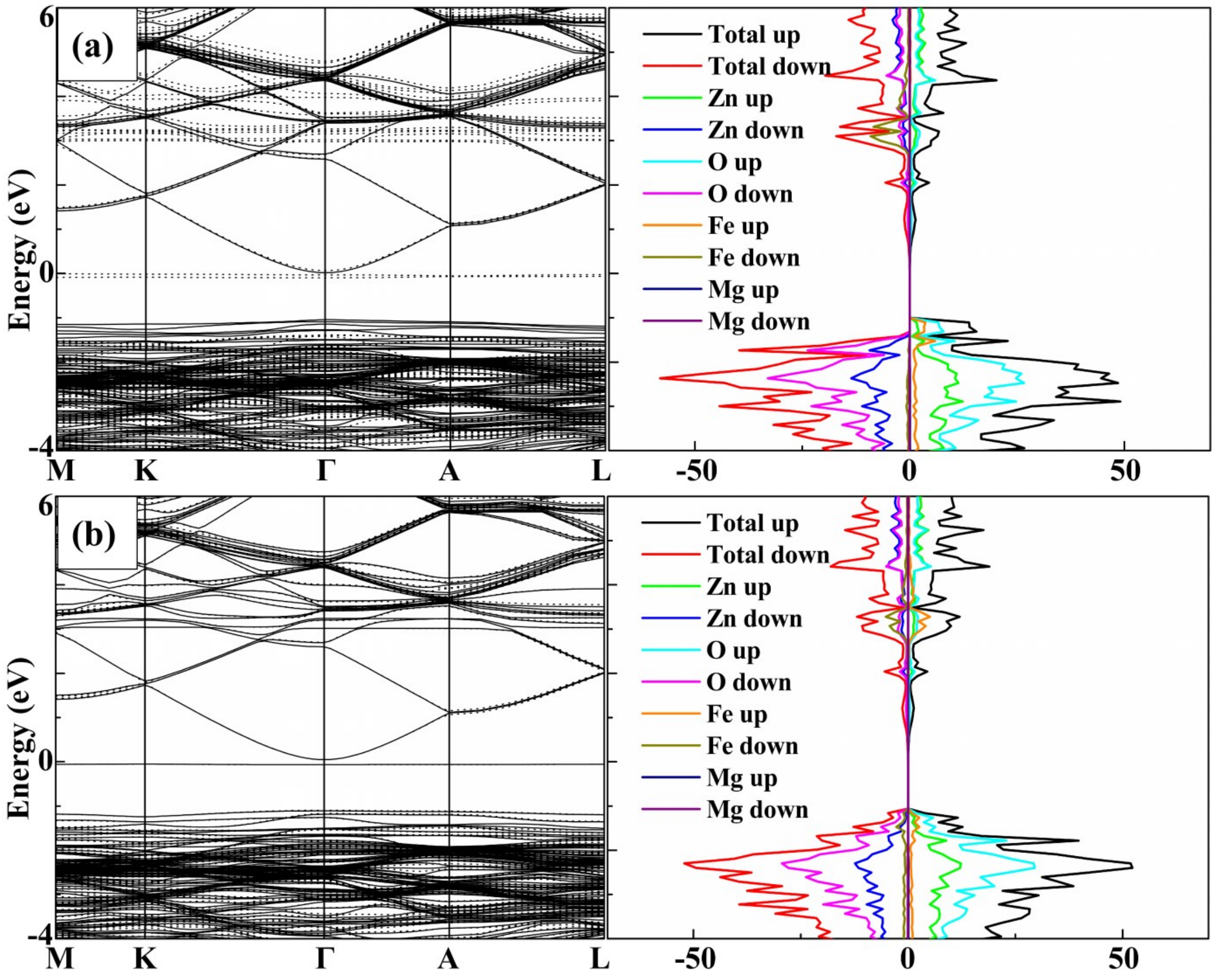

Figure S6. Band structure and density of state of configuration 6, the spin-up (solid line) and the spin-down (dotted line) channels and the corresponding density of state of 2Fe-doped MgZnO with (a) ↑↑, (b) ↑↓.

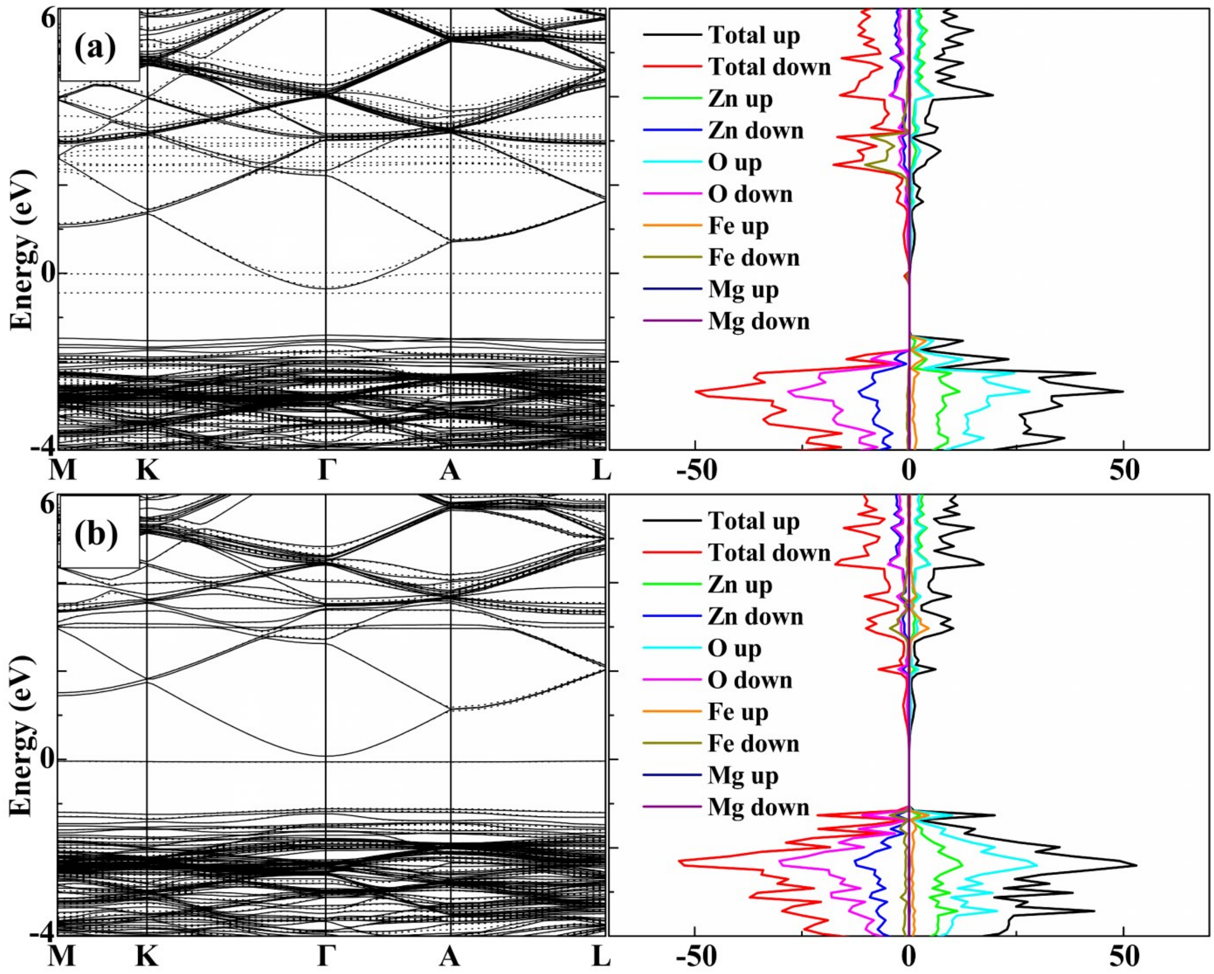

Figure S7. Band structure and density of state of configuration 7, the spin-up (solid line) and the spin-down (dotted line) channels and the corresponding density of state of 2Fe-doped MgZnO with (a) ↑↑, (b) ↑↓.

S4 The lattice constants of configuration 1 calculated by different U parameter of Zn

To explore the influence of different *U* parameters on the calculation, we also calculated the band structure and density of state of configuration 1. In Table S2 and Figure S8, the lattice

constant of configuration 1 calculated by different U parameters is presented, it is shown that a, b, c all decrease with the increasing of U parameter of Zn, and those calculated by U=3.8 is closer to the experimental result.

Table S2. The lattice constants of configuration 1 calculated by different U parameter of Zn.

U		3.8	6	8	10
↑↑	*a* = *b* (Å)	3.251	3.221	3.186	3.137
	c (Å)	5.231	5.184	5.126	5.045
↑↓	*a* = *b* (Å)	3.254	3.221	3.185	3.140
	c (Å)	5.230	5.184	5.125	5.047

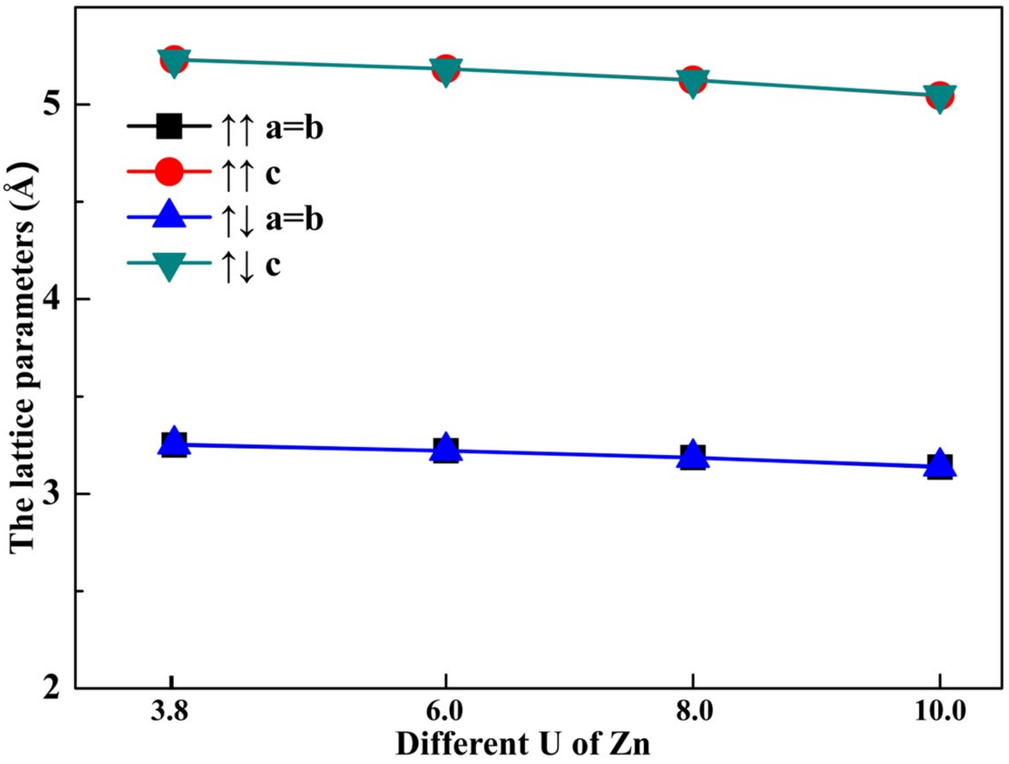

Figure S8. The lattice constants of configuration 1 calculated by different U parameter of Zn.

S5 The band gaps and density of state of configuration 1 calculated by different U parameter of Zn

In Table S3 and Figure S9 (the same as Figure 6 in the main text), the band gap of different spin channels with different *U* parameters of Zn are presented. It is shown that the band gap widens with the increase of *U* parameter. Similar to the results calculated by U=3.8, when the initial magnetic moment is ↑↑, the band gap calculated by different Us are also widen in the opposite channel. And when the initial magnetic moment is ↑↓, the band gap calculated by different Us are also narrow in both channels. The band structure and density of state calculated by different U parameters (U=3.8, 6, 8, 10) of Zn are shown in Figures S10-S13.

Table S3. The band gaps of configuration 1 calculated by different U parameter of Zn.

U		3.8	6	8	10
↑↑	Up	0.93	1.09	1.34	1.66
	Down	1.42	1.74	2.07	2.50
↑↓	Up	1.18	1.48	1.75	2.10
	Down	1.22	1.45	1.75	2.04

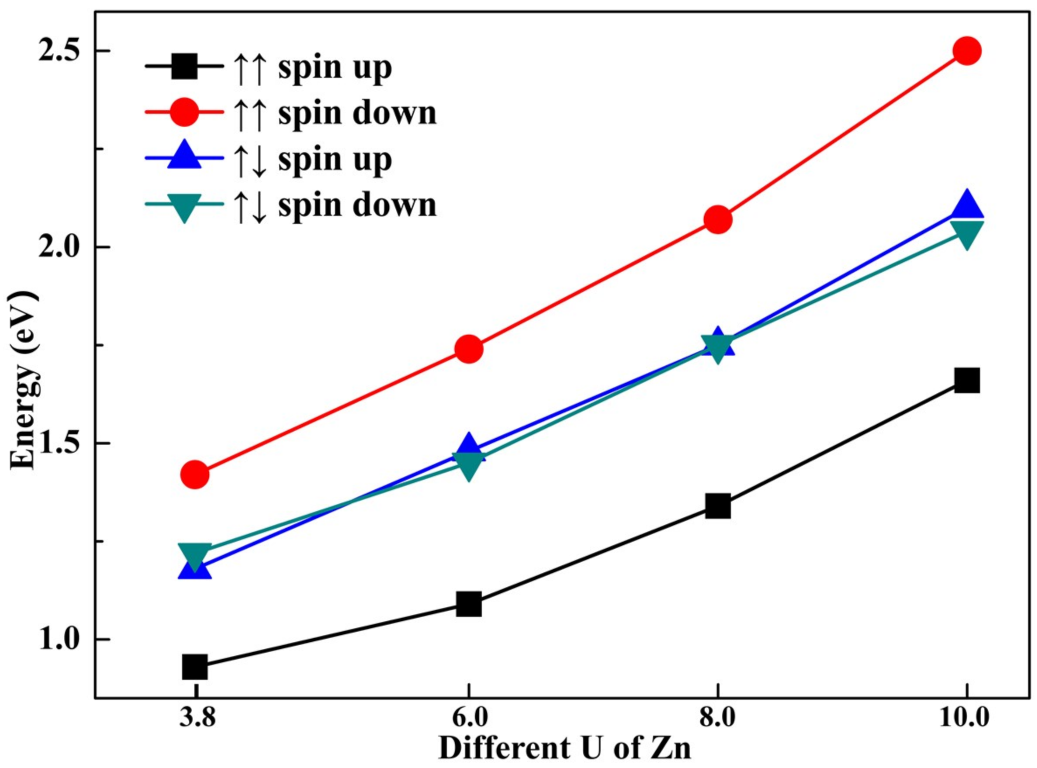

Figure S9. The band gaps of configuration 1 calculated by different U parameter of Zn.

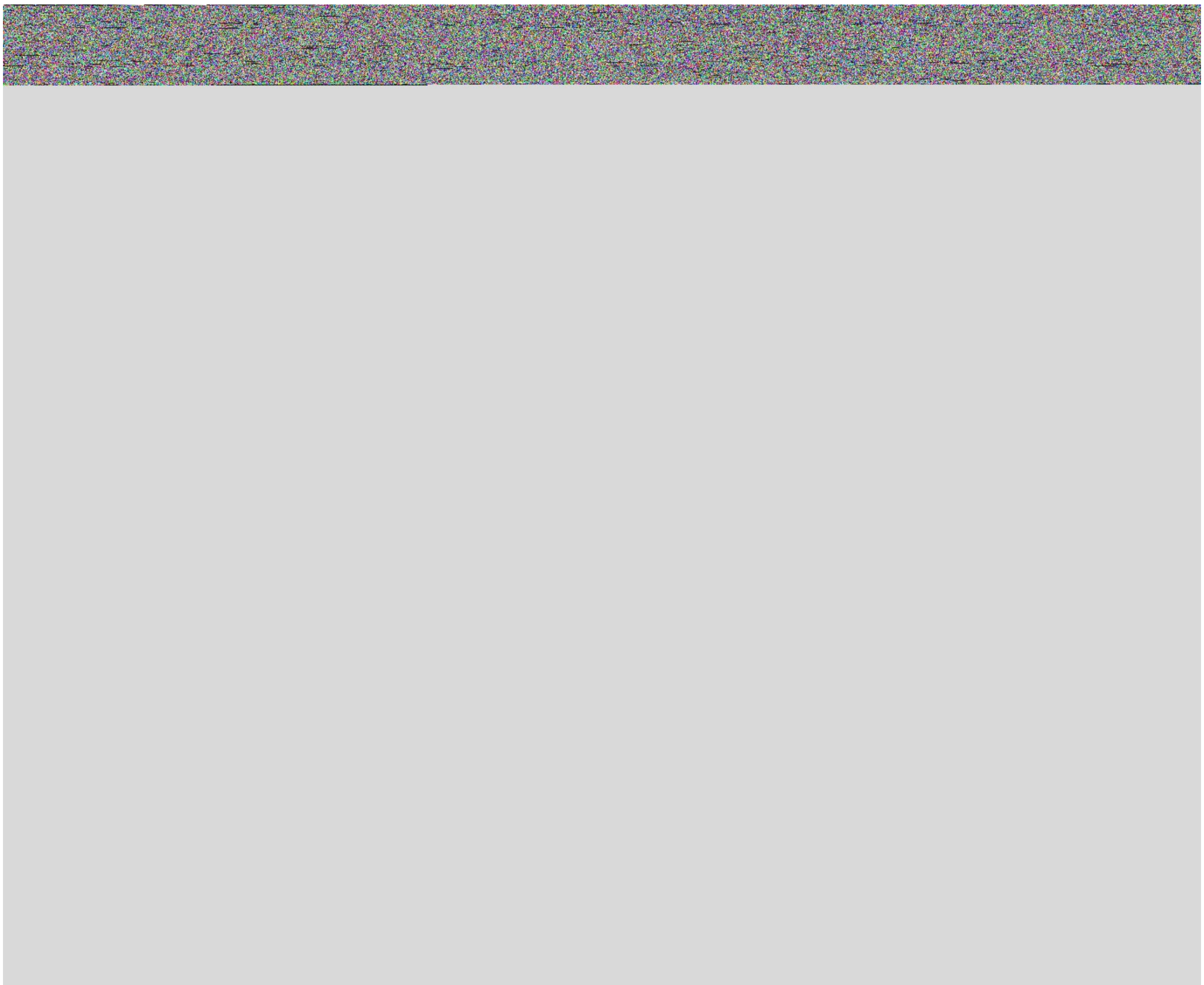

Figure S10. Band structure and density of state of configuration 1 calculated with U=3.8 of Zn, the spin-up (solid line) and the spin-down (dotted line) channels and the corresponding density of state of 2Fe-doped MgZnO with (a) ↑↑, (b) ↑↓.

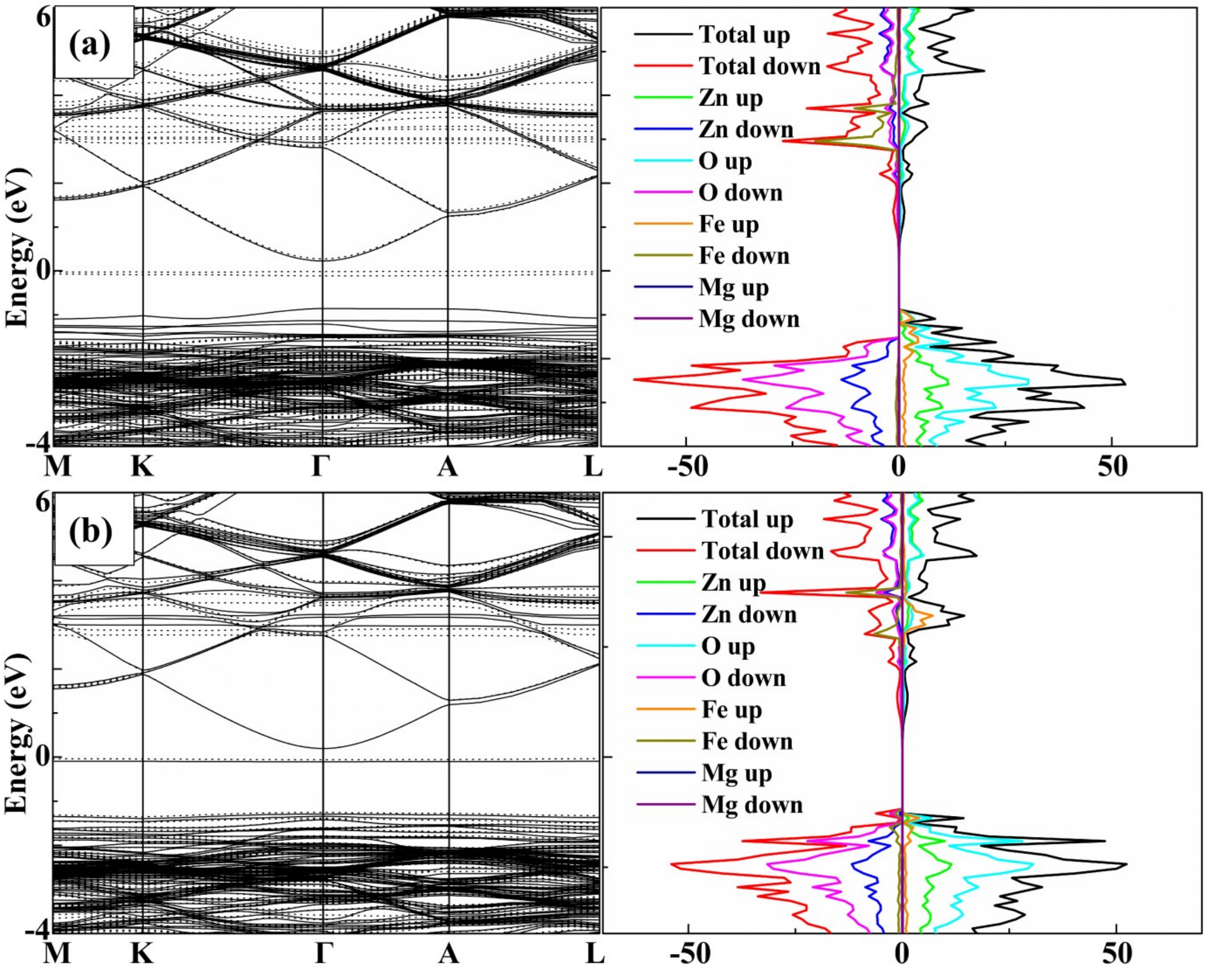

Figure S11. Band structure and density of state of configuration 1 calculated with U=6 of Zn, the spin-up (solid line) and the spin-down (dotted line) channels and the corresponding density of state of 2Fe-doped MgZnO with (a) ↑↑, (b) ↑↓.

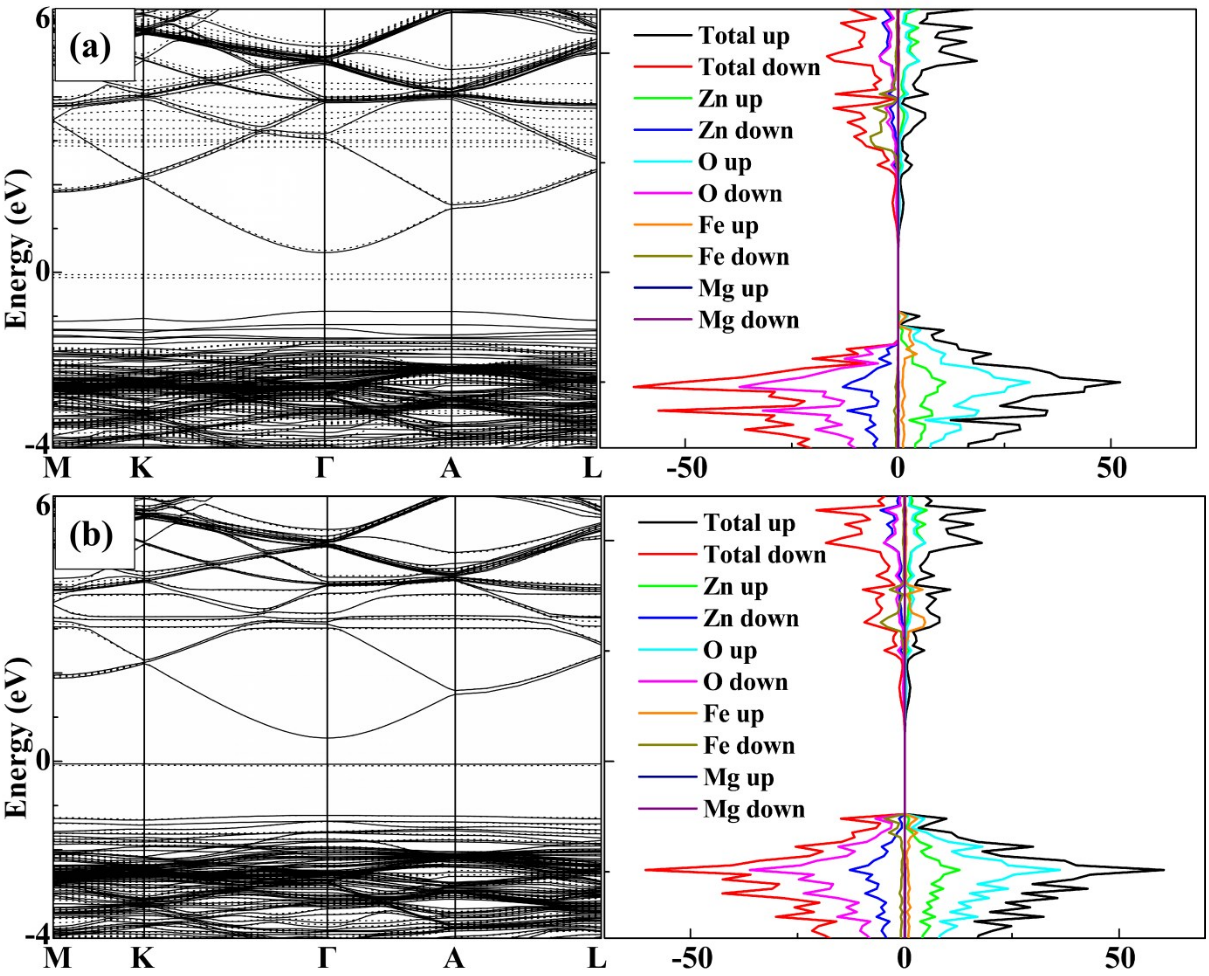

Figure S12. Band structure and density of state of configuration 1 calculated with U=8 of Zn, the spin-up (solid line) and the spin-down (dotted line) channels and the corresponding density of state of 2Fe-doped MgZnO with (a) ↑↑, (b) ↑↓.

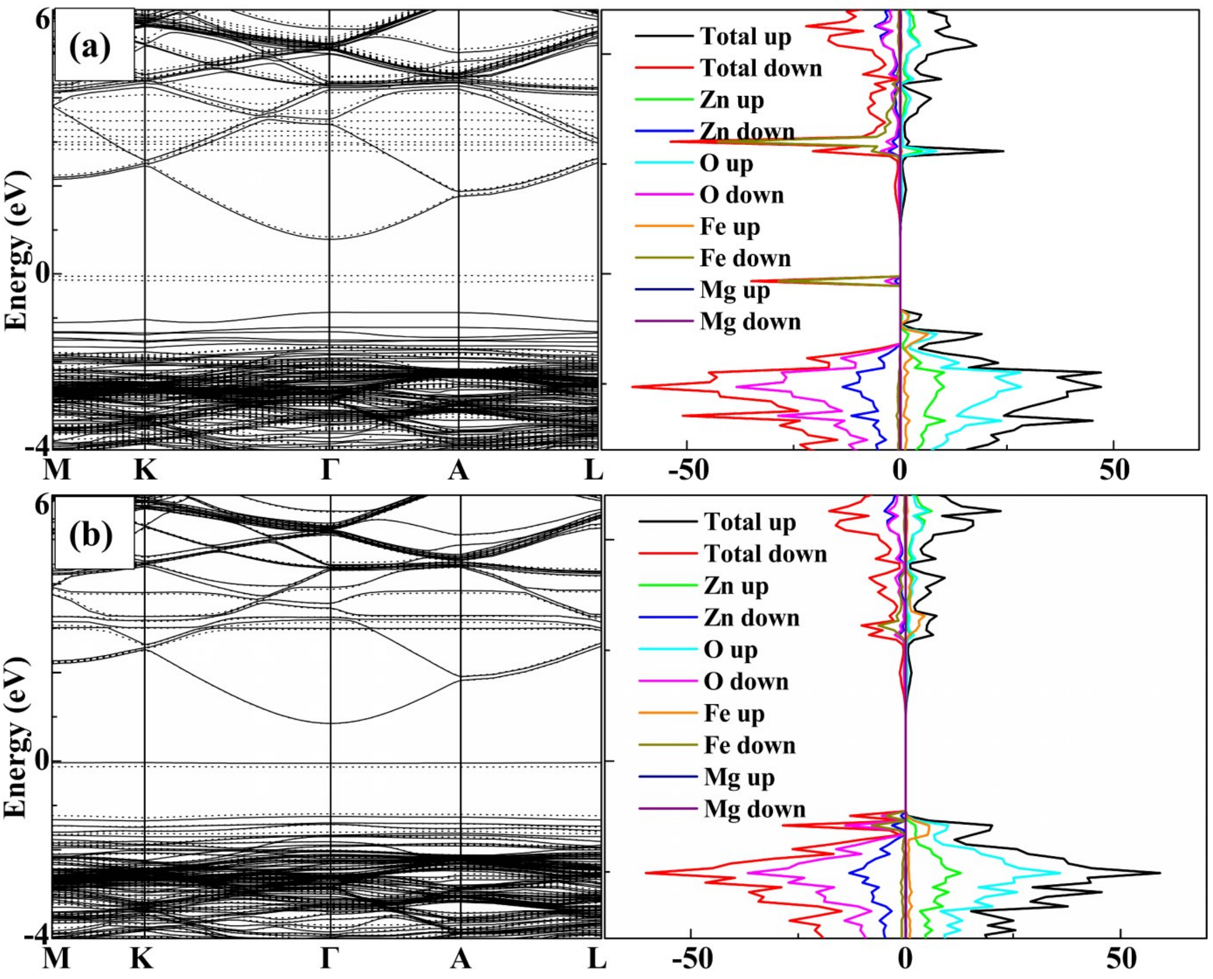

Figure S13. Band structure and density of state of configuration 1 calculated with U=10 of Zn, the spin-up (solid line) and the spin-down (dotted line) channels and the corresponding density of state of 2Fe-doped MgZnO with (a) ↑↑, (b) ↑↓.
